# Evaluating the Reporting Transparency and Interpretability of OLGA and OLGIM Staging in Gastric Cancer Risk Stratification

**DOI:** 10.14309/ctg.0000000000001068

**Published:** 2026-06-25

**Authors:** Jing Tian, Wenjie Xu, Shaofen Xu, Tingying Tang, Yimin Kang, Qiaoyu Peng, Fengbin Liu, Shaogang Huang, Xinlin Chen, Zhengkun Hou

**Affiliations:** 1 The First Clinical Medical College, Guangzhou University of Chinese Medicine, Guangzhou, China;; 2 The First Affiliated Hospital of Guangzhou University of Chinese Medicine, Guangzhou, China;; 3 College of Basic Medicine, Guangzhou University of Chinese Medicine, Guangzhou, China;; 4 The First Affiliated Hospital of Guangzhou University of Chinese Medicine, Yuexi Branch (Maoming Dianbai District Chinese Medicine Hospital), Maoming, China.

**Keywords:** gastric cancer, risk stratification, OLGA, OLGIM, interpretability, reporting transparency

## Abstract

**INTRODUCTION:**

Operative Link on Gastritis Assessment (OLGA) and Operative Link on Gastric Intestinal Metaplasia Assessment (OLGIM) are widely used for gastric cancer risk stratification, but published studies vary in how clearly their implementation is reported. We assessed the interpretability of OLGA/OLGIM implementation reporting, focusing on reporting transparency rather than methodological correctness, study quality, or guideline adherence.

**METHODS:**

We searched PubMed, Embase, and Cochrane CENTRAL from inception to March 20, 2026, with forward and backward citation tracking in Web of Science. Eligible studies applied OLGA and/or OLGIM and reported staging results. Two reviewers independently performed study selection and data extraction. Reporting interpretability was appraised using a prespecified framework based on 2 core components: biopsy sampling strategy and histological assessment with grading approach and stage derivation.

**RESULTS:**

Among 155 included studies, 75 (48.4%) had high reporting interpretability, 72 (46.5%) had moderate interpretability, and 8 (5.2%) had low interpretability. Although staging outcomes were widely reported, key details needed to understand stage generation were often incomplete, particularly biopsy sampling, grading approach, and stage derivation. These gaps limited the interpretability, reproducibility, and comparability of published OLGA/OLGIM evidence.

**DISCUSSION:**

Studies applying OLGA and OLGIM show substantial variability in implementation reporting. Limited interpretability often reflects incomplete reporting rather than demonstrable deficiencies in underlying methods. More consistent and transparent reporting may improve reproducibility, facilitate cross-study comparison, and support clinical translation.

## Graphical abstract

**Figure:**
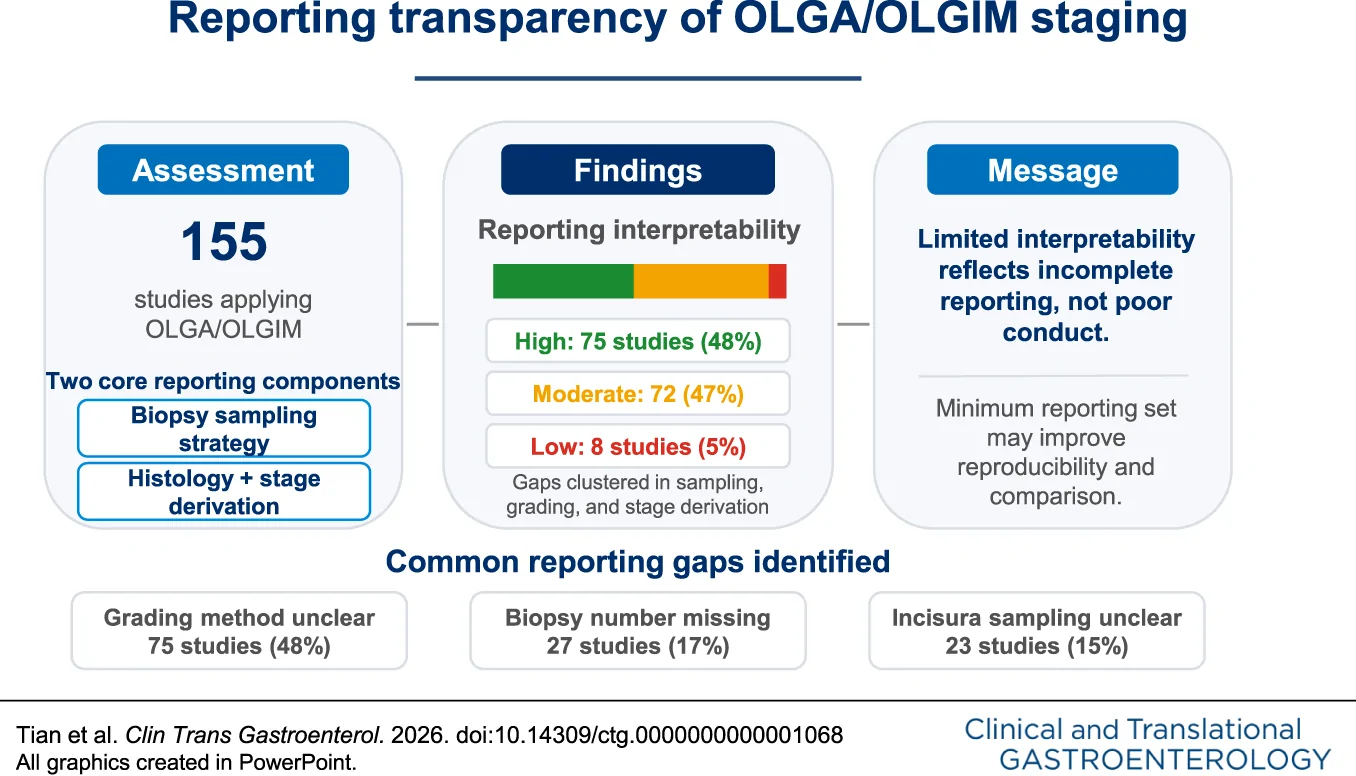

## INTRODUCTION

Gastric cancer remains a major and largely preventable global health challenge. Although age-standardized incidence rates have decreased in some regions, the absolute disease burden remains high, with an estimated 969,000 new cases and 660,000 deaths in 2022 (1), and a disproportionate burden in Eastern Asia and Eastern Europe (2). Addressing this challenge requires a shift from late-stage treatment to earlier recognition and management of gastric precancerous conditions. The Correa cascade describes the stepwise progression from nonatrophic chronic gastritis to atrophic gastritis, intestinal metaplasia (IM), dysplasia, and ultimately invasive gastric adenocarcinoma (3). *Helicobacter pylori*, classified as a class I carcinogen by the World Health Organization, is recognized as a major driver of this sequence (4), although other etiologies, including autoimmune gastritis, may also contribute to gastric mucosal atrophy and cancer risk (5). In this context, chronic atrophic gastritis and IM represent key precancerous conditions (6,7). A central clinical challenge is therefore not only detecting these lesions, but also stratifying risk in a way that can guide surveillance and intervention (8,9).

The Operative Link on Gastritis Assessment (OLGA) and the Operative Link on Gastric Intestinal Metaplasia Assessment (OLGIM) staging systems were introduced as structured frameworks for histology-based gastric cancer risk stratification (10,11). These systems translate histopathological findings into clinically relevant risk categories by integrating information on the severity and topographic distribution of atrophy or IM. Their interpretation depends on sufficient information about how biopsy sampling, histological assessment, and stage derivation were conducted and reported (11–13). Details on biopsy sites, number of specimens, grading approaches, compartment-specific assessment, and derivation of final stages are important for readers to understand how OLGA/OLGIM results were generated (11). When these details are incompletely reported, the resulting stage assignments may be difficult to interpret, compare, or reproduce, even if appropriate procedures were used in the original study.

OLGA and OLGIM have been incorporated into major international guidelines (8,9,14), including those from the European Society of Gastrointestinal Endoscopy, the European Helicobacter and Microbiota Study Group, and the American Gastroenterological Association, as reference frameworks for risk stratification and management. However, the interpretability of the evidence base depends not only on the use of these staging systems but also on the clarity with which their implementation is reported. To date, systematic evaluations focusing specifically on the transparency and completeness of OLGA/OLGIM implementation reporting remain limited. Published studies may differ in how they describe biopsy sampling strategies, histological grading methods, and the procedures used to derive final OLGA/OLGIM stages. Such variability in implementation reporting may limit reproducibility, cross-study comparability, and the integration of evidence into clinical practice.

A systematic evaluation of OLGA/OLGIM implementation reporting is therefore needed to clarify how interpretable the existing literature is. In this study, we assessed the transparency and completeness of reporting across published studies applying OLGA and/or OLGIM. Our objective was not to judge methodological correctness, study quality, or guideline adherence but to evaluate whether the published information was sufficient to understand how OLGA/OLGIM staging was implemented. By identifying common gaps in reporting, this study aimed to inform future reporting standards and support more reproducible, comparable, and clinically useful research on histology-based gastric cancer risk stratification.

## METHODS

The study was reported in accordance with the Preferred Reporting Items for Systematic Reviews and Meta-Analyses (PRISMA) 2020 statement whenever possible (15).

### Literature identification and study selection

We conducted a structured literature search in PubMed, Embase, and the Cochrane Central Register of Controlled Trials (CENTRAL) from database inception to March 20, 2026. Search terms included “atrophic gastritis,” “intestinal metaplasia,” “operative link on gastritis assessment,” “OLGA,” “operative link on gastric IM assessment,” and “OLGIM.” In addition, backward and forward citation tracking was performed in Web of Science to identify studies that cited the seminal articles. The complete search strategies for all databases are provided in the Supplementary Appendix (see Supplementary Digital Content, http://links.lww.com/CTG/B562).

Eligible studies were original human investigations that applied OLGA and/or OLGIM to assess gastritis severity or gastric cancer risk and reported OLGA/OLGIM staging results. Studies were excluded if OLGA or OLGIM was only mentioned without reporting staging results, or if the staging system was used solely for screening, eligibility determination, or background description rather than as an evaluative study component. Nonhuman studies and nonoriginal publications, including reviews, editorials, letters, conference abstracts, and case reports, were also excluded, as were non-English language studies.

No restrictions were imposed on the underlying etiology of gastritis. Studies involving *H. pylori*–associated gastritis, autoimmune gastritis, or mixed/unspecified etiologic contexts were eligible if they otherwise met the inclusion criteria.

### Data collection and extraction

All retrieved records were imported into EndNote 20, and duplicates were removed before screening. Titles and abstracts were screened independently by 2 investigators (J.T. and W.X.), followed by full-text review of potentially eligible articles. Data extraction was then performed independently by both investigators using a predefined standardized extraction form.

Extracted data items included the following: (i) basic study characteristics (title, authors, publication year, country, study design, and sample size); (ii) staging system used (OLGA, OLGIM, or both); (iii) biopsy sampling strategy; (iv) descriptions of histological grading approach and stage derivation process; (x) reporting of staging results; and (vi) additional contextual variables, such as *H. pylori* status and pathology-related quality control, and whether the incisura angularis was included in biopsy sampling, when available. Operational definitions for all extracted variables were predefined to ensure consistency and are detailed in Supplementary Table S3 (see Supplementary Digital Content, http://links.lww.com/CTG/B562). Detailed characteristics and extracted reporting variables for all included studies are presented in Supplementary Table S1 (see Supplementary Digital Content, http://links.lww.com/CTG/B562).

Disagreements were resolved by consensus, with adjudication by a third investigator (Z.H.) when necessary. Inter-reviewer agreement for study selection and data extraction was quantified using the Cohen kappa statistics.

### Reporting interpretability framework

**Figure 1.**
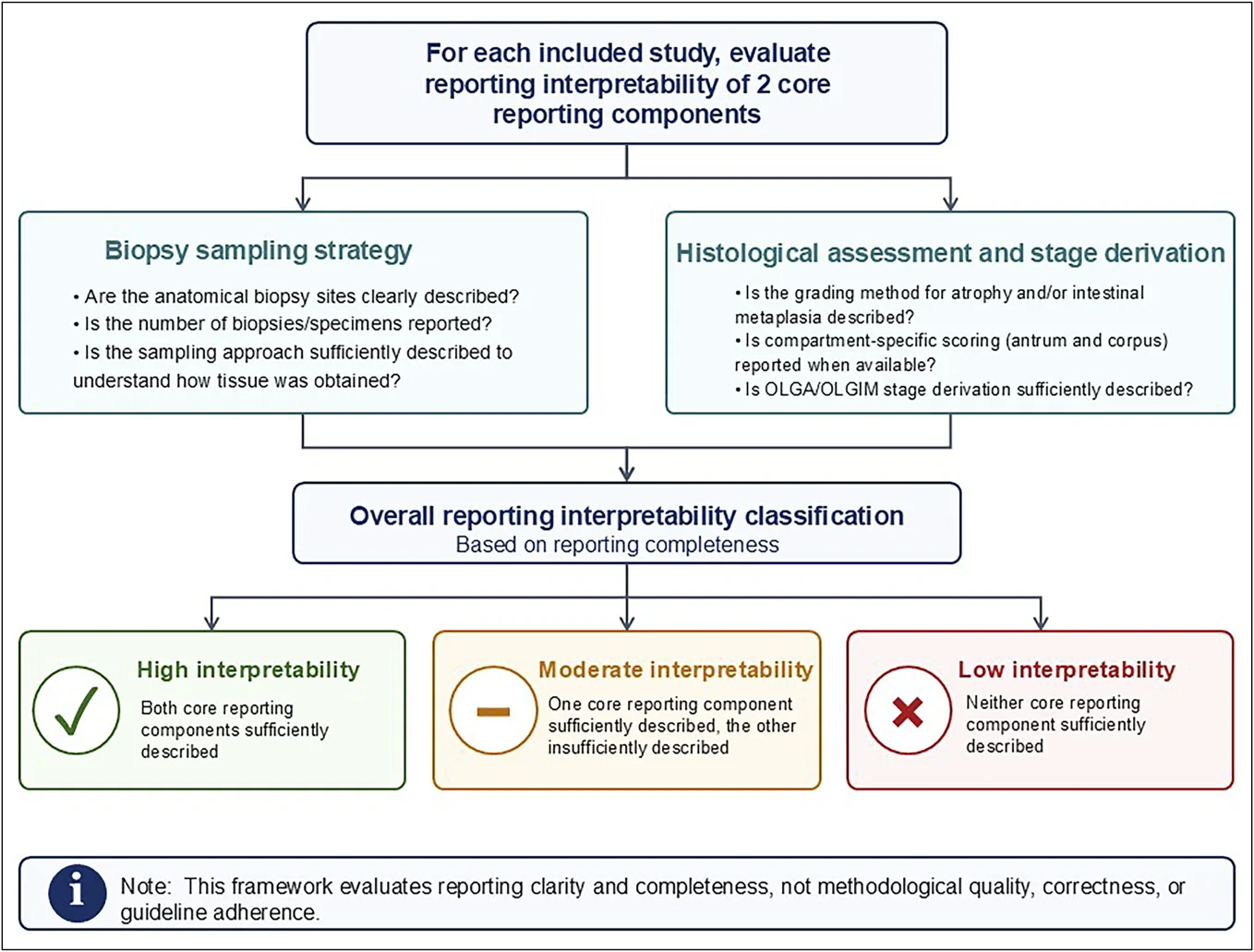
Prespecified framework for classifying reporting interpretability of OLGA/OLGIM implementation in included studies. Studies were classified as having high, moderate, or low reporting interpretability according to reporting completeness for 2 core components: biopsy sampling strategy and histological assessment with stage derivation. The framework evaluated reporting clarity, not methodological correctness, study quality, or guideline adherence. OLGA, Operative Link on Gastritis Assessment; OLGIM, Operative Link on Gastric Intestinal Metaplasia Assessment.

To evaluate how clearly published studies reported the implementation of OLGA/OLGIM staging systems, we developed a 3-level reporting interpretability framework (Figure 1). The framework focused on the transparency and completeness of reporting, not on methodological correctness, study quality, or adherence to guideline recommendations.

Two core reporting components were evaluated for each study: (i) biopsy sampling strategy, defined as whether the anatomical biopsy sites and number of biopsies or specimens were clearly reported and (ii) histological assessment and stage derivation, defined as whether sufficient information was reported on grading of atrophy and/or IM, compartment-specific assessment, and derivation of OLGA/OLGIM stages.

Histological grading was considered sufficiently reported if either quantitative (e.g., a 0–3 scale based on percentage ranges with thresholds such as no atrophy = 0%, mild atrophy = 1%–30%, moderate atrophy = 31%–60%, and severe atrophy >60%) or semiquantitative (e.g., a visual analog scale with 0 = absent, 1 = mild, 2 = moderate, and 3 = severe) methods were described. Alternatively, qualitative grading (e.g., mild/moderate/severe) was accepted provided the grading method was explicitly stated. In addition, explicit reporting of compartment-specific scoring (i.e., separate assessment of gastric antrum and corpus) was documented as part of the staging description when available.

Studies were categorized into 3 reporting interpretability levels based on the completeness of reporting across the 2 core components. High reporting interpretability indicated that both core components were sufficiently reported, allowing readers to understand how OLGA/OLGIM stages were generated. Moderate reporting interpretability indicated that one core component was sufficiently reported, whereas the other was incompletely reported, allowing only partial understanding of OLGA/OLGIM implementation. Low reporting interpretability indicated that neither core component was sufficiently reported, limiting meaningful interpretation of how OLGA/OLGIM stages were derived.

Importantly, insufficient published detail was not interpreted as evidence that the corresponding procedures were omitted in the original studies. Instead, it was interpreted as limited reporting within the published article. The classification was used only to stratify reporting interpretability and was not intended to appraise study quality, methodological correctness, or procedural adherence. Additional variables, including *H. pylori* status and pathology-related quality control measures, were recorded as contextual information and were not used to assign reporting interpretability levels.

### Assessment of guideline-referenced studies

In addition to the primary analysis, we examined the reporting interpretability of studies cited in clinical practice guidelines. We searched PubMed and Embase from January 2005 to April 17, 2026, to identify guidelines or consensus documents on the management or surveillance of gastric precancerous conditions that explicitly referenced OLGA and/or OLGIM. The guideline search strategies are provided in the Supplementary Appendix (see Supplementary Digital Content, http://links.lww.com/CTG/B562).

For each guideline, we extracted recommendation statements citing OLGA/OLGIM and their supporting references. These references were cross-checked against the studies included in the primary data set, and studies that overlapped with the primary data set were classified using the same reporting interpretability framework.

### Outcomes and statistical analysis

All included studies were analyzed descriptively to characterize overall patterns in OLGA/OLGIM use and implementation reporting. Secondary analyses summarized reporting of the 2 core reporting components, namely biopsy sampling strategy and histological assessment with stage derivation. Reported biopsy sampling practices, including the number and location of biopsies when available, were summarized descriptively based on information provided in each study, but specific sampling patterns were not used to assign reporting interpretability levels. We also descriptively summarized the reporting interpretability of guideline-cited studies. Detailed results are presented in Supplementary Table S2 (see Supplementary Digital Content, http://links.lww.com/CTG/B562).

## RESULTS

### Search results and study characteristics

**Figure 2.**
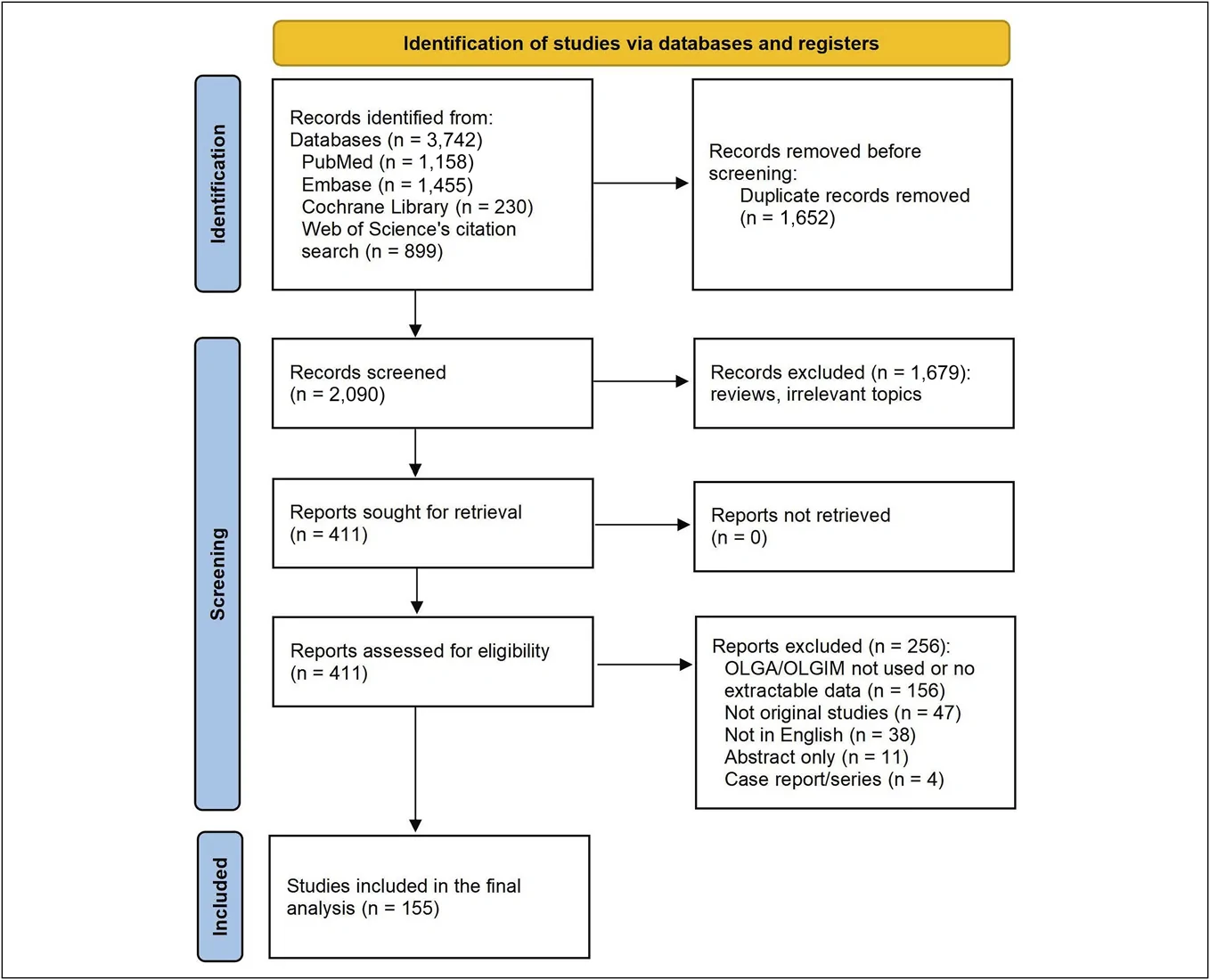
Flow diagram of study selection. Records identified through database searching and citation tracking were screened according to predefined eligibility criteria. The final analysis included 155 original human studies applying OLGA and/or OLGIM. OLGA, Operative Link on Gastritis Assessment; OLGIM, Operative Link on Gastric Intestinal Metaplasia Assessment.

A total of 2,090 records were screened, and 411 full-text articles were assessed for eligibility. Ultimately, 155 studies met the inclusion criteria and were included in the final analysis (Figure 2). Inter-reviewer agreement was high, with the Cohen kappa values of 0.871 for study selection and 0.848 for data extraction.

The included studies were published between 2007 and 2025 and originated from 34 countries or regions, with China contributing the largest proportion (n = 39, 25.2%). Study designs were predominantly cross-sectional (n = 89, 57.4%) or cohort (n = 42, 27.1%) studies. Sample sizes ranged from 23 to 7,945 participants. Regarding staging-system use, 93 studies (60.0%) applied both OLGA and OLGIM, 47 (30.3%) applied OLGA alone, and 15 (9.7%) applied OLGIM alone. Characteristics of the 155 included studies are summarized in Table 1. Detailed study-level information on OLGA/OLGIM implementation reporting is provided in Supplementary Table S1 (see Supplementary Digital Content, http://links.lww.com/CTG/B562).

**Table 1.** Characteristics of included studies (n = 155)

Characteristic	Category	n (%)
Study design	Cross-sectional	89 (57.4)
	Cohort	42 (27.1)
	Case-control	15 (9.7)
	Other	9 (5.8)
Geographic region	Asia	79 (51.0)
	Europe	61 (39.4)
	Other^a^	15 (9.7)
Staging system used	Both OLGA and OLGIM	93 (60.0)
	OLGA only	47 (30.3)
	OLGIM only	15 (9.7)
Reporting interpretability	High	75 (48.4)
	Moderate	72 (46.5)
	Low	8 (5.2)
Sample size	Median (range)	259 (23–7,945)

n = number of included studies; % = percentage relative to total 155 studies; OLGA = Operative Link on Gastritis Assessment; OLGIM = Operative Link on Gastric Intestinal Metaplasia Assessment.

a Includes studies from the Americas (n = 10), Africa (n = 1), and multinational collaborations (n = 4), as well as any study spanning multiple UNSD regions (United Nations Statistics Division geoscheme). Percentages may not sum to 100% because of rounding.

### Overall reporting interpretability classification

Of the 155 included studies, 75 (48.4%) were classified as having high reporting interpretability, 72 (46.5%) as moderate reporting interpretability, and 8 (5.2%) as low reporting interpretability. Thus, 147 studies (94.8%) provided at least some interpretable information on OLGA/OLGIM implementation, although nearly half did not completely report both core reporting components.

**Figure 3.**
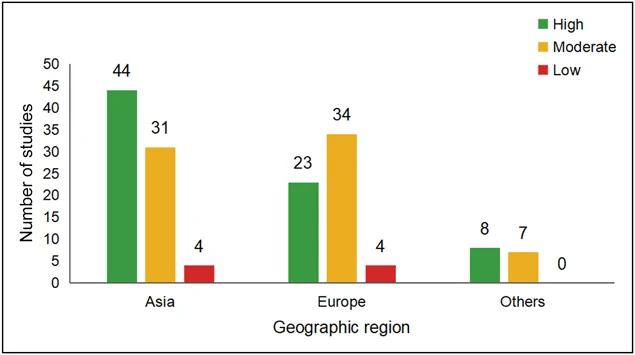
Regional distribution of reporting interpretability levels among included studies. Bars show the number of studies classified as having high, moderate, or low reporting interpretability by geographic region. Other regions include studies from the Americas, Africa, and multinational collaborations.

Among studies conducted in Asia, 55.7% were classified as having high reporting interpretability and 5.1% as low reporting interpretability. Among studies conducted in Europe, 55.7% were classified as having moderate reporting interpretability, whereas 37.7% were classified as having high reporting interpretability. Studies from other regions were distributed between high and moderate reporting interpretability, although the number of studies was limited (Figure 3).

### Reporting of core OLGA/OLGIM implementation details

Among the 155 studies, grading of atrophy and/or IM was reported using a 0–3 percentage-based scale in 18 studies (11.6%), a 0–3 semiquantitative scale in 53 studies (34.2%), and qualitative descriptions in 9 studies (5.8%). The grading method was not clearly reported in 75 studies (48.4%).

Compartment-specific assessment, defined as separate assessment of the antrum and corpus, was reported in 149 studies (96.1%). Consistent with the eligibility criteria, OLGA/OLGIM staging results were available in all studies. *H. pylori* status was reported in 147 studies (94.8%), and pathology-related quality control measures were described in 88 studies (56.8%). The number of biopsy specimens was specified in 128 studies (82.6%) and was not reported in 27 studies (17.4%). In addition, 74 studies (47.7%) explicitly reported inclusion of at least one biopsy from the incisura angularis, 58 studies (37.4%) reported sampling protocols that did not include the incisura, and 23 studies (14.8%) did not provide sufficient information to determine whether the incisura was sampled.

### Reporting interpretability of guideline-referenced studies

Among the studies included in the primary data set, 42 were cited by clinical guidelines (see Supplementary Table S2, Supplementary Digital Content, http://links.lww.com/CTG/B562). The reporting interpretability of these guideline-referenced studies varied: 26 studies (61.9%) were classified as having high reporting interpretability, 13 (31.0%) as moderate reporting interpretability, and 3 (7.1%) as low reporting interpretability. Table 2 summarizes the reporting interpretability classification and guideline citation counts for guideline-cited studies, whereas Supplementary Table S2 (see Supplementary Digital Content, http://links.lww.com/CTG/B562) provides the underlying guideline-reference mapping and study-level identifiers.

**Table 2.** Guideline citation counts and interpretability levels of included studies

Cited study (author, year)	Number of citing guidelines	Reporting interpretability
Capelle et al. (11), 2010	11	High
Rugge, 2010	7	High
Rugge, 2007	4	High
Rugge, 2019	4	Moderate
Isajevs, 2014a	4	High
Rugge, 2018	4	High
Rugge et al. (24), 2011a	4	High
den Hollander, 2018	4	High
Satoh, 2008	3	High
Marcos, 2020	3	Moderate
Zhou, 2016	3	High
Lee, 2022	3	Moderate
Cho, 2013	3	High
Kim, 2017	2	High
Marcos-Pinto, 2012	2	High
den Hoed, 2013	2	High
Kodama, 2013	2	High
Varbanova, 2016	2	High
Lahner, 2015	2	High
Rugge et al. (13), 2008	2	High
Rugge, 2011b	2	Moderate
Na, 2023	2	High
Tsai, 2013	2	High
Isajevs, 2014b	2	High
Quach, 2011	2	Moderate
Castro, 2019	1	Moderate
Cai, 2021	1	Moderate
Rugge, 2012	1	Moderate
Zhang, 2019	1	Moderate
Tun, 2021	1	Moderate
Esposito, 2021	1	Low
Conti, 2021	1	Low
Daugule, 2011	1	High
Fassan, 2012	1	Moderate
Chapelle, 2020	1	High
Vannella, 2010	1	Moderate
Sun, 2022	1	Moderate
Ferrari, 2023	1	High
Huang, 2025	1	High
Wang, 2017	1	High
Khomeriki, 2023	1	Low
Yun, 2018	1	High

**Figure 4.**
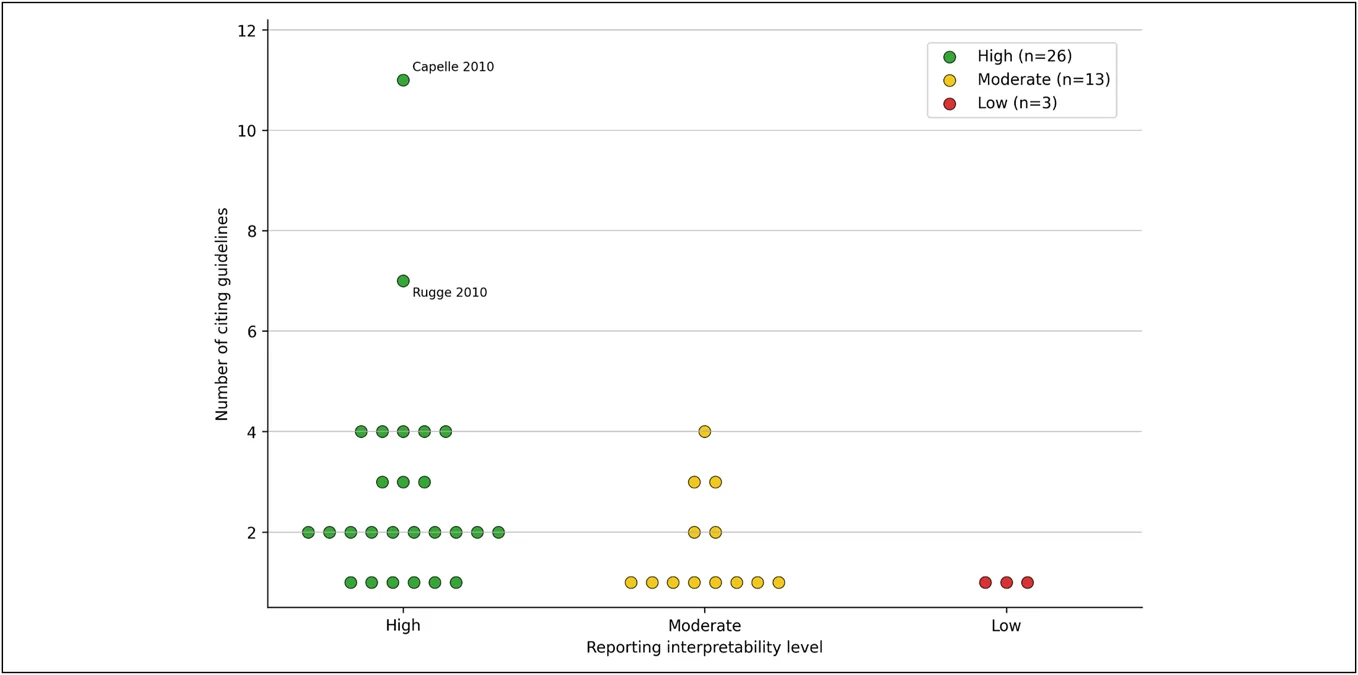
Guideline citation frequency by reporting interpretability level among guideline-cited studies. Each point represents one guideline-cited study. Points with identical citation counts are displayed in a beeswarm-like arrangement. Only studies cited by ≥ 5 guidelines are labeled; full study-level details are provided in Table 2 and Supplementary Table S2 (see Supplementary Digital Content, http://links.lww.com/CTG/B562).

Figure 4 shows the distribution of guideline citation counts across reporting interpretability levels. Citation frequency was not consistently aligned with reporting interpretability. The most frequently cited study, Capelle 2010 (11), was referenced by 11 guidelines and was classified as having high reporting interpretability. However, several other guideline-cited studies with moderate or low reporting interpretability were also cited in guidelines, whereas some studies with high reporting interpretability had relatively few guideline citations. Overall, guideline-cited studies showed variable reporting completeness, indicating that guideline citation frequency should not be interpreted as a direct marker of reporting interpretability.

## DISCUSSION

### Principal findings and the gap between reporting and interpretability

This study provides, to our knowledge, the first systematic evaluation of reporting interpretability in published studies applying OLGA and OLGIM staging. Although these systems were developed to standardize histology-based gastric cancer risk stratification and are endorsed by major international guidelines (2,8,16,17), their implementation was reported with substantial variability across studies. In many studies, final OLGA/OLGIM stages were reported, but the information needed to understand how those stages were generated was incomplete. Reporting gaps were most evident for biopsy sampling strategy, grading approach, and stage derivation, whereas compartment-specific assessment and *H. pylori* status were documented in most studies.

A key implication is that limited reporting interpretability should not be equated with poor conduct in the original studies. In many cases, the limitation lies in published documentation rather than in demonstrable problems with OLGA/OLGIM application. Nevertheless, incomplete reporting may constrain independent interpretation, reproducibility, and cross-study comparability (18). Improving reporting transparency may therefore enhance the usefulness of OLGA/OLGIM-based evidence. Recent guideline updates support a more nuanced view of OLGA/OLGIM implementation. European recommendations emphasize adequate topographic sampling of the antrum/incisura and corpus with separate labeling, while allowing biopsies from the incisura angularis to be optional in selected settings (19,20). These considerations suggest that the main gap identified in this review is not necessarily between guideline recommendations and actual practice, but between the conceptual clarity of OLGA/OLGIM and the variable transparency of their reporting.

The framework also identified variability within the same reporting interpretability level. For example, Bornschein et al. (21) provided detailed histological grading information but did not clearly describe the biopsy sampling strategy, whereas Miftahussurur et al. (22) used a simplified 2-site biopsy protocol but clearly reported sampling sites and specimen numbers. This contrast illustrates that reporting transparency, rather than conformity to a specific biopsy protocol, determined interpretability in this review. A nonstandard sampling approach may still be highly interpretable if it is clearly reported, whereas a standard protocol may be less interpretable if key details are not described.

### Sources of reporting variability

The sources of limited reporting interpretability can be understood across 3 sequential steps in OLGA/OLGIM implementation: biopsy sampling, histological grading, and stage derivation.

First, at the sampling level, incomplete or variable reporting of biopsy protocols may limit reproducibility. The central issue in this review was not whether specific biopsy sites were sampled, but whether the sampling procedure was described with sufficient clarity to allow readers to understand and potentially replicate it. This distinction is particularly important because recent evidence and guideline updates suggest that some aspects of biopsy sampling, including the role of incisura biopsies, may be context dependent rather than universally fixed. Such variability may also reflect real-world implementation challenges, including differences in endoscopic workflow, biopsy labeling practices, coordination between endoscopy and pathology units, local resource availability, and procedure time. These considerations are consistent with recent guideline emphasis on adequate topographic sampling and separate specimen labeling (19,20). These factors, however, could not be verified from published reports.

Second, at the histological assessment level, reporting of grading approaches was often incomplete or variable. Some studies reported percentage-based or threshold-informed approaches, whereas others reported qualitative or semiquantitative categorizations (23). These approaches may not be interchangeable and could lead to different stage assignments despite similar histological severity (24). In addition, variation in the reporting of atrophy grading may further limit comparability across studies, particularly when only qualitative descriptors such as mild, moderate, or severe were provided (23). Previous work has shown higher interobserver agreement for IM than for atrophy (12), which may partly explain the greater reproducibility of OLGIM compared with OLGA (24).

Third, at the stage derivation level, many studies reported final OLGA or OLGIM stages without clearly describing how antral and corpus findings were integrated. Without this information, readers may not be able to determine whether stage derivation followed the conventional matrix, a modified approach, or an implicit local practice. This is important because final staging results are only fully interpretable when the preceding sampling and grading information can be linked to the derivation of the reported stage. Taken together, these findings indicate that reporting interpretability depends on cumulative transparency across biopsy sampling, histological grading, and stage derivation. Given the inherent subjectivity in histopathology (12), transparent reporting is important for making published staging results interpretable, reproducible, and comparable.

### Clinical and research implications

The observed variability in reporting has important implications for evidence synthesis and clinical translation. When key implementation details are incompletely reported, it becomes difficult to distinguish true biological variation from differences in biopsy sampling, grading approach, or stage derivation. This may limit the interpretation of pooled evidence and complicate systematic reviews, meta-analyses, and guideline development. Incomplete reporting may also reduce the ability of readers to judge whether differences in reported OLGA/OLGIM stage distributions reflect population-level risk, clinical setting, or differences in how staging was implemented.

In the guideline-cited subset, citation frequency was not consistently aligned with reporting completeness (Figure 4). This finding should be interpreted cautiously, because guideline citation is influenced by multiple factors, including clinical relevance, sample size, study design, historical importance, and availability of outcome data. Nevertheless, it suggests that reporting completeness may deserve more explicit consideration during evidence appraisal, particularly when studies are used to support surveillance recommendations or risk stratification strategies.

At the clinical level, our findings do not argue against the continued use of OLGA and OLGIM. Rather, they emphasize that the value of these systems depends in part on transparent reporting of how they are applied. Clear reporting of biopsy sampling, grading approach, and stage derivation can help clinicians and researchers interpret stage assignments in context, compare findings across settings, and identify whether apparent differences across studies reflect clinical heterogeneity or differences in implementation reporting.

### Limitations

Despite these contributions, several limitations should be considered. First, our assessment relied exclusively on published reports. Consequently, the actual procedures used in the original studies could not always be verified when implementation details were incompletely documented (25). This limitation reflects the focus of our study, which was to evaluate reporting interpretability rather than methodological adequacy. Moreover, the reporting interpretability framework was developed as a prespecified analytical framework for this review and should not be regarded as an established mandatory standard for OLGA/OLGIM reporting.

Second, the included studies spanned heterogeneous clinical settings, populations, and endoscopic indications, which may have contributed to genuine variability in biopsy practice and reporting style. The geographic variation observed in reporting interpretability levels may reflect differences in reporting traditions, journal requirements, or resource availability. However, firm conclusions are limited by the small number of studies from non-Asian and non-European regions.

Third, some studies may have included patients with corpus-restricted atrophic patterns suggestive of autoimmune gastritis, even when this was not explicitly stated. This may affect comparability across cohorts. Finally, our analysis addressed the interpretability of published reporting rather than the validity, prognostic performance, or clinical utility of OLGA/OLGIM themselves.

### Future directions and system evolution

Future work should prioritize the development of a practical minimum reporting set for studies using OLGA, OLGIM, or related gastric precancerous staging systems. Such a reporting set should include biopsy location and labeling, number of specimens, grading approach, handling of intermediate values, compartment-specific assessment, and derivation of final stage assignments. A concise and feasible checklist may improve transparency without imposing excessive reporting burden. Future studies, including expert consensus or prospective validation studies, are needed to determine which reporting elements are most feasible, clinically relevant, and necessary for a minimum reporting set.

Complementary strategies may further refine gastric cancer risk stratification, but they do not remove the need for transparent reporting. Noninvasive biomarkers, such as serum pepsinogen-based approaches and combined *H. pylori* serology and pepsinogen testing, may help identify lower-risk individuals and optimize endoscopic resource allocation (26,27). Endoscopy-based approaches may also enhance stratification when high-quality visualization guides targeted sampling (28). The Endoscopic Grading of Gastric Intestinal Metaplasia provides real-time estimation of metaplastic burden and may improve efficiency and reproducibility (29,30). However, because Endoscopic Grading of Gastric Intestinal Metaplasia relies on systematic evaluation of predefined gastric regions, incomplete or uneven assessment may introduce limitations similar to those observed in histology-based staging.

Artificial intelligence-assisted digital histopathology represents another promising direction. Objective quantification of IM or atrophy may reduce observer variability and improve consistency when integrated into structured workflows (31–33). Nevertheless, artificial intelligence-assisted tools should be viewed as complements to clear reporting of sampling strategies, grading approaches, and stage derivation, not as substitutes for it. Prospective multicenter validation remains necessary before widespread clinical implementation (34).

Several refinements to existing staging approaches have also been proposed. Combined or modified frameworks incorporating both atrophy and IM, such as the Operative Link on Gastric Intestinal Metaplasia and Atrophy, may improve risk discrimination in selected populations (35). These approaches warrant further validation. However, our findings suggest that improving reporting transparency is a more immediate and broadly applicable priority than replacing existing staging systems.

The literature on OLGA and OLGIM demonstrates substantial variability in the reporting of core implementation details underlying stage assignment. Although staging outcomes are widely reported, incomplete description of biopsy sampling, grading approach, and stage derivation often limits interpretability, reproducibility, and comparability.

Improving reporting transparency, rather than replacing existing staging systems, represents a practical next step. Clearer documentation of OLGA/OLGIM implementation may strengthen evidence synthesis and support more reliable translation of gastric precancerous staging into clinical decision-making and future guideline development.

**Study Highlights**

**WHAT IS KNOWN**

✓ Operative Link on Gastritis Assessment (OLGA) and Operative Link on Gastric Intestinal Metaplasia Assessment (OLGIM) are widely used for gastric cancer risk stratification.
✓ Their application relies on histological assessment and structured stage assignment.
✓ Variation in OLGA/OLGIM implementation reporting across studies may affect interpretability and comparability.

**WHAT IS NEW HERE**

✓ This study identifies variability in how OLGA/OLGIM implementation is reported in published studies.
✓ Differences were mainly observed in the reporting of biopsy sampling, grading approaches, and stage assignment.
✓ The findings may help inform more transparent and consistent reporting in future OLGA/OLGIM research.

## CONFLICTS OF INTEREST

**Guarantor of the article:** Zhengkun Hou, MD.

**Specific author contributions:** J.T.: conceptualization, data extraction, manuscript writing. W.X.: data extraction, manuscript editing. S.X.: data extraction, review and analysis of the methodology. T.T.: data collection and quality assurance. Y.K.: literature search and manuscript editing. Q.P.: statistical analysis and manuscript revision. F.L.: supervision, manuscript editing. S.H.: supervision, review of the manuscript. X.C.: statistical guidance and manuscript review. Z.H.: conceptualization, study design, data interpretation, manuscript finalization. All authors have approved the final version of the manuscript.

**Financial support:** This study was funded by the National Key Research and Development Program of China (No. 2023YFC3503002); the Young Backbone Talent Cultivation Project of the First Affiliated Hospital of Guangzhou University of Chinese Medicine (No. Zhongyi Yiyuan [2023] 186); the Guangdong Basic and Applied Basic Research Foundation Youth Project (No. 2023A1515110731); the Guangdong Basic and Applied Basic Research Foundation (Grant No. 2021A1515220045); and the Guangzhou University of Chinese Medicine School-Hospital Joint Scientific and Technological Innovation Fund Project (No. GZYFS2024Y06).

**Potential competing interests:** The authors declare no potential competing interests.

**IRB Approval Statement:** As this article is based solely on existing literature and does not involve original data collection or human subject research, no IRB approval is needed.

## Supplementary Material

## ABBREVIATIONS:

AGA: American Gastroenterological Association
AI: artificial intelligence
AIG: autoimmune gastritis
CENTRAL: Cochrane Central Register of Controlled Trials
EGGIM: Endoscopic Grading of Gastric Intestinal Metaplasia
EHMSG: European Helicobacter and Microbiota Study Group
ESGE: European Society of Gastrointestinal Endoscopy
*H. pylori*: *Helicobacter pylori*
IM: intestinal metaplasia
OLGA: operative link on gastritis assessment
OLGIM: operative link on gastric intestinal metaplasia assessment
PRISMA: preferred reporting items for systematic reviews and meta-analyses
