# Supplementary material for: Evaluating the Reporting Transparency and Interpretability of OLGA and OLGIM Staging in Gastric Cancer Risk Stratification
Source: Clin Transl Gastroenterol. 2026 Jun 25;17(9):e01068. doi: 10.14309/ctg.0000000000001068 (PMC13593235; doi:10.14309/ctg.0000000000001068)
Supplement: Supplementary file 1 [file ct9-17-e01068-s001.docx]

# Supplementary Appendix

Contents

[Supplementary Appendix	1](#_Toc13989)

[Detailed Search Strategy for Primary Studies	2](#_Toc25526)

[Search Strategy for Primary Study Identification	2](#_Toc19126)

[PubMed Search Strategy	2](#_Toc32333)

[Embase Search Strategy	2](#_Toc20533)

[Central Search Strategy	3](#_Toc24874)

[Web of Science Search Strategy	3](#_Toc29744)

[Detailed Search Strategy for Clinical Practice Guidelines	4](#_Toc7067)

[Search Strategy for Guideline Identification	4](#_Toc11606)

[PubMed Search Strategy	4](#_Toc9183)

[Embase Search Strategy	4](#_Toc12809)

# Detailed Search Strategy for Primary Studies

Search Strategy for Primary Study Identification

- **Databases: PubMed, Embase, Cochrane Central Register of Controlled Trials (CENTRAL), Web of Science**
- **Date Range: Inception to March 20, 2026**
- **Date of Search: March 20, 2026**

## **PubMed Search Strategy**

| No | Query | Records |
| --- | --- | --- |
| #1 | "OLGA"[Title/Abstract] OR "OLGIM"[Title/Abstract] OR "OLGA staging"[Title/Abstract] OR "OLGIM staging"[Title/Abstract] OR "Operative Link on Gastritis Assessment"[Title/Abstract] OR "Operative Link for Gastritis Assessment"[Title/Abstract] OR "Operative Link on Gastric Intestinal Metaplasia Assessment"[Title/Abstract] OR "Operative Link for Gastric Intestinal Metaplasia Assessment"[Title/Abstract] | 446 |
| #2 | "Gastritis, Atrophic"[Mesh] OR "atrophic gastritis"[Title/Abstract] OR "chronic atrophic gastritis"[Title/Abstract] OR "gastric atrophy"[Title/Abstract] OR ("intestinal metaplasia"[Title/Abstract] AND gastric[Title/Abstract]) OR "gastric intestinal metaplasia"[Title/Abstract] OR "gastric premalignant lesion*"[Title/Abstract] OR "gastric precancerous lesion*"[Title/Abstract] OR "gastric precancerous condition*"[Title/Abstract] OR "gastric preneoplastic lesion*"[Title/Abstract] | 10,679 |
| #3 | "risk stratif*"[Title/Abstract] OR "risk prediction"[Title/Abstract] OR "gastric cancer risk"[Title/Abstract] OR "surveill*"[Title/Abstract] | 399,266 |
| #4 | #2 AND #3 | 870 |
| #5 | (#1 OR #4) NOT ("case reports"[Publication Type] OR "letter"[Publication Type] OR "comment"[Publication Type] OR "editorial"[Publication Type]) | 1,158 |

## **Embase Search Strategy**

| No | Query | Records |
| --- | --- | --- |
| #1 | OLGA:ti,ab,kw OR OLGIM:ti,ab,kw OR 'OLGA staging':ti,ab,kw OR 'OLGIM staging':ti,ab,kw OR 'operative link on gastritis assessment':ti,ab,kw OR 'operative link for gastritis assessment':ti,ab,kw OR 'operative link on gastric intestinal metaplasia assessment':ti,ab,kw OR 'operative link for gastric intestinal metaplasia assessment':ti,ab,kw | 1,141 |
| #2 | 'atrophic gastritis'/exp OR 'gastric atrophy'/exp OR ('intestinal metaplasia'/exp AND 'stomach'/exp) OR 'atrophic gastritis':ti,ab,kw OR 'chronic atrophic gastritis':ti,ab,kw OR 'gastric atrophy':ti,ab,kw OR ('intestinal metaplasia':ti,ab,kw AND gastric:ti,ab,kw) OR 'gastric intestinal metaplasia':ti,ab,kw OR 'gastric premalignant lesion*':ti,ab,kw OR 'gastric precancerous lesion*':ti,ab,kw OR 'gastric precancerous condition*':ti,ab,kw OR 'gastric preneoplastic lesion*':ti,ab,kw | 19,998 |
| #3 | 'risk stratification':ti,ab,kw OR 'risk prediction':ti,ab,kw OR 'gastric cancer risk':ti,ab,kw OR surveill*:ti,ab,kw | 570,757 |
| #4 | #2 AND #3 | 1,962 |
| #5 | (#1 OR #4) NOT ('letter'/it OR 'editorial'/it OR 'note'/it OR 'erratum'/it OR 'conference abstract'/it OR 'conference paper'/it OR 'conference review'/it) | 1,455 |

## **Central Search Strategy**

| No | Query | Records |
| --- | --- | --- |
| #1 | OLGA OR OLGIM OR "OLGA staging" OR "OLGIM staging" OR "Operative Link on Gastritis Assessment" OR "Operative Link for Gastritis Assessment" OR "Operative Link on Gastric Intestinal Metaplasia Assessment" OR "Operative Link for Gastric Intestinal Metaplasia Assessment" | 171 |
| #2 | atrophic gastritis OR chronic atrophic gastritis OR gastric atrophy OR intestinal metaplasia OR gastric intestinal metaplasia OR gastric premalignant lesions OR gastric precancerous lesions OR gastric precancerous conditions OR gastric preneoplastic lesions | 1,217 |
| #3 | "risk stratification" OR "risk prediction" OR "gastric cancer risk" OR surveill* | 17,627 |
| #4 | #2 AND #3 | 136 |
| #5 | (#1 OR #4) in Trials | 230 |

## **Web of Science Search Strategy**

Citation tracking was performed in the Web of Science Core Collection (SCI-EXPANDED) on March 20, 2026, using both forward (citing articles) and backward (reference list) approaches.

| **No.** | **Seed Publication** | **Forward citations**  **(n)** | **Backward references**  **(n)** |
| --- | --- | --- | --- |
| 1 | Rugge M, Genta RM. *Staging gastritis: an international proposal* (2005) | 127 | 5 |
| 2 | Rugge M, Correa P, Di Mario F, et al. *OLGA staging for gastritis: a tutorial* (2008) | 211 | 31 |
| 3 | Capelle LG, de Vries AC, Haringsma J, et al. *The staging of gastritis with the OLGA system by using intestinal metaplasia as an accurate alternative for atrophic gastritis* (2010) | 380 | 35 |
| 4 | Rugge M, Fassan M, Pizzi M, et al. *Operative link for gastritis assessment vs operative link on intestinal metaplasia assessment* (2011) | 94 | 16 |

# Detailed Search Strategy for Clinical Practice Guidelines

Search Strategy for Guideline Identification

- **Databases: PubMed, Embase**
- **Date Range: January 1, 2005 – April 17, 2026**
- **Date of Search: April 17, 2026**

## **PubMed Search Strategy**

("Practice Guideline"[Publication Type] OR "Guideline"[Publication Type] OR "Guideline"[Title/Abstract] OR "consensus"[Title/Abstract]) AND ("Gastritis"[MeSH Terms] OR "gastritis, atrophic"[MeSH Terms] OR "Stomach Neoplasms"[MeSH Terms] OR ("intestinal metaplasia"[Title/Abstract] OR "gastric intestinal metaplasia"[Title/Abstract])) AND 2005/01/01:2026/04/17[Date - Publication]

Results: ****1,343 records****

## **Embase Search Strategy**

| **No** | **Query** | **Records** |
| --- | --- | --- |
| #1 | gastritis:ti OR 'gastritis atrophic':ti OR 'stomach neoplasms':ti OR 'intestinal metaplasia':ti OR 'gastric intestinal metaplasia':ti | 15,748 |
| #2 | 'guideline':ab,kw,ti OR 'consensus':ab,kw,ti | 520,688 |
| #3 | #1 AND #2 | 163 |
| #4 | #3 AND [2005-2026]/py | 145 |

Results: ****145 records****
